# High spectral purity Kerr frequency comb radio frequency photonic oscillator

**DOI:** 10.1038/ncomms8957

**Published:** 2015-08-11

**Authors:** W. Liang, D. Eliyahu, V. S. Ilchenko, A. A. Savchenkov, A. B. Matsko, D. Seidel, L. Maleki

**Affiliations:** 1 OEwaves Inc., 465 North Halstead Street, Suite 140, Pasadena, California 91107, USA

## Abstract

Femtosecond laser-based generation of radio frequency signals has produced astonishing improvements in achievable spectral purity, one of the basic features characterizing the performance of an radio frequency oscillator. Kerr frequency combs hold promise for transforming these lab-scale oscillators to chip-scale level. In this work we demonstrate a miniature 10 GHz radio frequency photonic oscillator characterized with phase noise better than −60 dBc Hz^−1^ at 10 Hz, −90 dBc Hz^−1^ at 100 Hz and −170 dBc Hz^−1^ at 10 MHz. The frequency stability of this device, as represented by Allan deviation measurements, is at the level of 10^−10^ at 1–100 s integration time—orders of magnitude better than existing radio frequency photonic devices of similar size, weight and power consumption.

## Abstract (web summary)

An optical frequency comb demodulated on a photodiode can generate a radio frequency signal with high spectral purity at a frequency corresponding to the comb spacing. Here, Liang *et al.* demonstrate a frequency-comb-based radio frequency photonic oscillator characterized with low phase noise and high frequency stability.

## Graphical abstract

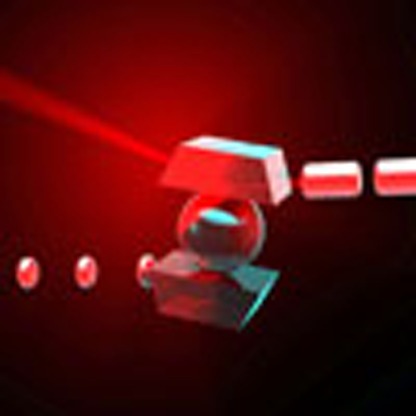

With the recent exponential growth of communications data, the available frequency bands are crowded, and the trend is to move up in frequency to increase the modulation bandwidth. This requirement places a severe challenge on the source of the carrier wave, namely the oscillator, as properties of this source determine the quality of a communication link consisting of a transmitter and a receiver^1^,^2^,^3^,^4^,^5^.

Information transmitted on a data link is carried as modulation of phase, frequency or amplitude of a carrier wave. Two characteristics of the carrier wave, frequency and spectral purity, determine the amount of information that can be communicated. The frequency of the carrier wave dictates the maximum modulation frequency, which in turn determines the amount of data that can be transmitted. Spectral purity of the carrier wave determines the number of distinct data channels that may be transmitted, and fixes the channel capacity of the link. This general constraint applies to all links in which a transmitter sends a signal, to be detected by a receiver, and includes data and communications links, radar, and any other link consisting of a sender and a receiver.

Electronic oscillators draw their spectral purity from the high-quality factor (Q) of the resonator in their circuit, which generally degrades with increasing frequency. To address this limitation, schemes to provide higher spectral purity at higher frequency typically involve more complex architectures to multiply low frequency but spectrally pure oscillator signals, to higher frequencies; or to design resonators for oscillators that directly produce higher frequency signals. These approaches are limited in efficacy, and typically add to the size, power and complexity of the signal generation subsystem.

In recent years, optical generation of spectrally pure radio frequency (RF) (microwave and mm-wave) signals has attracted considerable attention^6^. This is because available high-speed photodetectors can produce microwave and mm-wave frequencies from the beat produced by two (or more) phase-locked lasers, with their output frequency limited only by the bandwidth of the detector. In particular, the beat note generated with an optical frequency comb^7^ with multiple phase-locked harmonics, exhibits an extremely high spectral purity at a frequency corresponding to the comb spacing. The high spectral purity of such a photonic oscillator is the result of division of the noise of the optical source by a factor as large as the ratio of the optical to beat note frequencies.

Indeed, an RF photonic oscillator based on a femtosecond mode-locked laser has demonstrated the highest spectral purity at a microwave frequency^8^,^9^,^10^. In the case of electronic oscillators, the floor of the phase noise spectrum, representing the signal to noise ratio of the oscillator with white spectral characteristic, is typically limited by the thermal noise floor dictated by the noise of the amplifier in the oscillator loop. While most high performance amplifiers have low enough phase noise at high Fourier frequencies, the close to carrier noise of the oscillator is subject to degradation due to *f*^−1^ noise of the amplifier. Recent demonstration of high power uni-traveling carrier photodetectors allow generating RF outputs with 25 mW and higher with photonic oscillators, eliminating the noise associated with the electronic amplifier^10^.

Despite the success of femtosecond laser-based oscillators, their widespread utility outside the laboratory is limited because of the size of the laser and associated stabilization equipment and circuitry. The advent of microresonator-based lasers^11^,^12^,^13^,^14^,^15^,^16^,^17^ as well as hyper-parametric oscillators and Kerr frequency combs^18^,^19^,^20^,^21^,^22^,^23^,^24^,^25^,^26^,^27^,^28^,^29^,^30^,^31^,^32^,^33^,^34^ are an important development towards realization of photonic oscillators with small size and power requirements, for widespread use.

Highly spectrally pure signals at microwave and mm-wave frequencies have also been generated with multimode Raman^14^ and Brillouin lasers^11^,^12^,^16^,^17^ as the beat between the laser harmonics. A microresonator-based Raman laser was used to produce 35 GHz RF signals characterized with phase noise of −30 dBc Hz^−1^ at 100 Hz frequency offset and −140 dBc Hz^−1^ at 10 MHz and higher frequency offsets^14^. RF photonic oscillators based on Brillouin lasers have similar performance. For instance, a microresonator-based Brillouin laser was used to generate RF signals with phase noise of −30 dBc Hz^−1^ at 100 Hz offset, −90 dBc Hz^−1^ at 10 kHz offset, −110 dBc Hz^−1^ at 100 kHz offset and −160 dBc Hz^−1^ at 100 MHz and higher frequency offsets^16^. The carrier frequency of the RF signal, 21.7 GHz, was set by the properties of the resonator host material.

Kerr frequency comb oscillators based on whispering gallery mode (WGM) microresonators compete with laser-based devices. The typical Kerr comb oscillator includes a nonlinear microresonator pumped with continuous wave (cw) light, and a fast photodiode (PD) (Fig. 1). The microresonator transforms the cw light via hyper-parametric oscillation to a phase-locked frequency comb that generates spectrally pure RF signals by demodulation on a fast PD.

The extremely large Qs achievable with these resonators lead to generation of very high spectral purity RF signals with modest laser powers^19^,^22^,^23^,^30^,^31^,^35^,^36^. For example, optical comb-based 35 GHz oscillators with phase noise of −115 dBc Hz^−1^ at 10 kHz offset and −130 dBc Hz^−1^ at 100 kHz offset have been produced in a hybrid integrated package as small as the size of a dime (US ten-cent coin), with a semiconductor laser pumping a MgF_2_ microresonator^6^. Signals at 22 GHz RF characterized with phase noise of −113 dBc Hz^−1^ at 10 kHz offset frequency have been demonstrated with fused silica microresonators^35^.

Here our goal is to study limitations of Kerr comb-based RF photonic oscillators, as well as to improve their phase noise and stability performance. We demonstrate a Kerr comb oscillator that exhibits phase noise orders of magnitude lower than earlier realizations of WGM resonator-based devices. It also features a superior performance compared with chip-scale laser-based RF photonic oscillators. Its measured power spectral density of phase noise approaches the fundamental limit of the achievable performance, and is −60 dBc Hz^−1^ at 10 Hz, −90 dBc Hz^−1^ at 100 Hz and −170 dBc Hz^−1^ at 10 MHz. Furthermore, its frequency stability measured as Allan deviation approaches 10^−10^ at 1–100 s integration times. The oscillator consumes <25 mW of optical power, with its total power consumption <2 W, set by the power consumed for thermal stabilization of the package. This approach makes a large array of high performance applications, requiring superior performance in microwave and mm-wave frequency range, possible.

## Results

### Generating a broad Kerr frequency comb

We produced a Kerr comb with a high-Q MgF_2_ WGM resonator (see Methods section) to generate the oscillator signal. Crystalline MgF_2_ is suitable for manufacturing very high-Q WGM resonators because of its excellent mechanical and optical properties^37^,^38^,^39^,^40^. At wavelengths exceeding 1,440 nm, MgF_2_ has an anomalous group velocity dispersion and a relatively small thermo-refractive constant. Anomalous group velocity dispersion facilitates phase matching of the nonlinear process and reduces the threshold of hyper-parametric oscillation responsible for comb generation^20^,^25^. The small thermo-refractive constant of MgF_2_ increases the threshold of low frequency thermo-optical instabilities that hinder phase locking of the comb lines, so that highly efficient generation of Kerr frequency combs may be achieved in this wavelength range^32^,^41^,^42^,^43^.

**Figure 2 f2:**
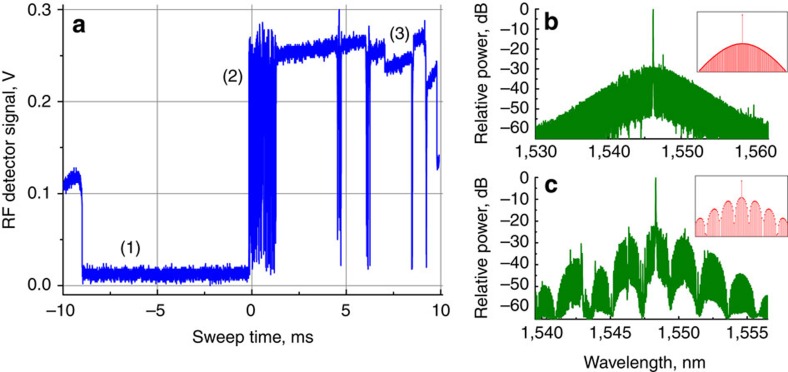
Illustration of the operation regimes of the oscillator. (**a**) Signal of the RF detector generated by the light leaving the resonator when the frequency of the laser is swept through the resonator modes. Area (1) corresponds to the case of no frequency comb with harmonics separated by a single FSR. Area (2) corresponds to chaotic regime. Area (3) corresponds to various comb attractors where each step stands for a different comb pattern. Panels (**b**) and (**c**) show examples of the combs generated in area (3). Insets in (**a**) and (**b**) stand for theoretically simulated comb patterns. The simulation was performed by solving a set of ordinary differential equations describing Kerr frequency comb generation^48^,^49^.

By pumping the MgF_2_ resonator with cw light we observed broad optical combs similar to those generating short optical pulses^43^ (Fig. 2), and studied the produced oscillation in RF frequency domain. Figure 2a illustrates dependence of the RF power generated by the light exiting the resonator on a fast PD. There is no RF signal when either the optical frequency comb is not generated, or a multi-FSR oscillation is present. Modification of the detuning results in appearance of a chaotic oscillation, followed by generation of phase-locked frequency combs. Multi-stable operation of the system with different comb envelopes is observed in this region. Examples of these combs are illustrated in Fig. 2b,c. A numerical simulation allows obtaining similar comb frequency patterns, shown in insets to Fig. 2b,c.

### Observing RF signals produced by the frequency comb

**Figure 3 f3:**
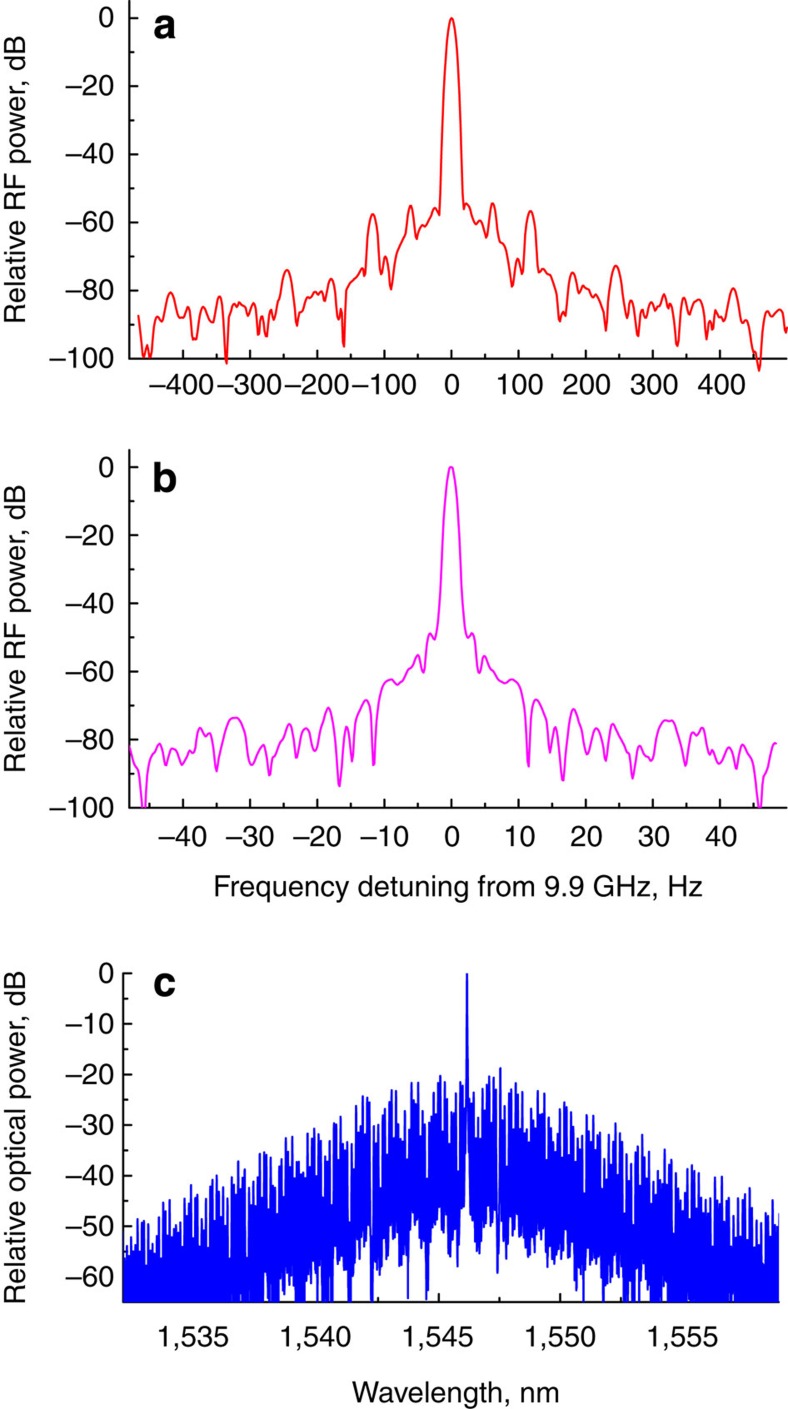
RF signal generated by the broad frequency comb. Power spectra of an RF frequency signal generated by a broad optical frequency comb on a fast PD (**a**,**b**) measured with different resolution bandwidth. (**a**) The spectrum is taken using an RF spectrum analyzer with 1 KHz span and 9 Hz resolution bandwidth. The smaller peaks are 60 Hz and its harmonics, and arise from the power source. (**b**) The spectrum is measured with 1 Hz resolution bandwidth by downconverting the RF signal to 8 MHz. The downconvertion is realized by mixing the 9.9 GHz signal produced by the comb with a signal of a stable RF synthesizer. The optical frequency comb generating the RF signal is illustrated by panel (**c**).

We studied the RF signal generated by one of the broad frequency combs on a fast PD (see Methods section) and observed an exceptionally high spectrally pure RF line, Fig. 3. The modulation sidebands visible in the spectrum result from cross-talk with the 60 Hz voltage source. The observed RF tone confirms that the Kerr frequency comb is promising for generation of spectrally pure RF signals. This kind of a photonic generator is attractive since it can be miniaturized as it does not need a high-Q RF cavity to produce the RF signal, and can be realized with chip-scale hybrid integration.

We introduced several optimization steps to significantly improve the oscillator performance as compared with previous attempts^19^,^22^,^23^,^30^,^31^,^35^,^36^. First, we stabilized the temperature of the MgF_2_ resonator to mK level. Second, we stabilized the temperature of the entire optical path to ensure that the relative noise between the laser and the resonator mode is minimized. These two improvements became possible owing to tight hybrid integration of the Kerr frequency comb oscillator. Third, we used an add-drop prism configuration (see Methods section) to reduce the impact of the laser noise on the RF noise^44^,^45^. Fourth, we used a narrow-band RF filter to replace the detector shot noise with thermal noise. Finally, we operated the broad-band comb at phase-locked regime to produce RF signals with improved close-in noise as compared with the signals originated from narrow-band combs used previously. We confirm the phase-locked nature of the comb and explain the observed high offset frequency noise behavior of the RF signals with numerical simulations. We prove that the close-in noise of the comb oscillator is determined by thermal fluctuations of the WGM resonator resulting from the laser noise, by showing a correlation between the phase noise of the pump laser and the RF signal (see Discussion section).

### Improving RF spectral purity using a drop optical port

**Figure 4 f4:**
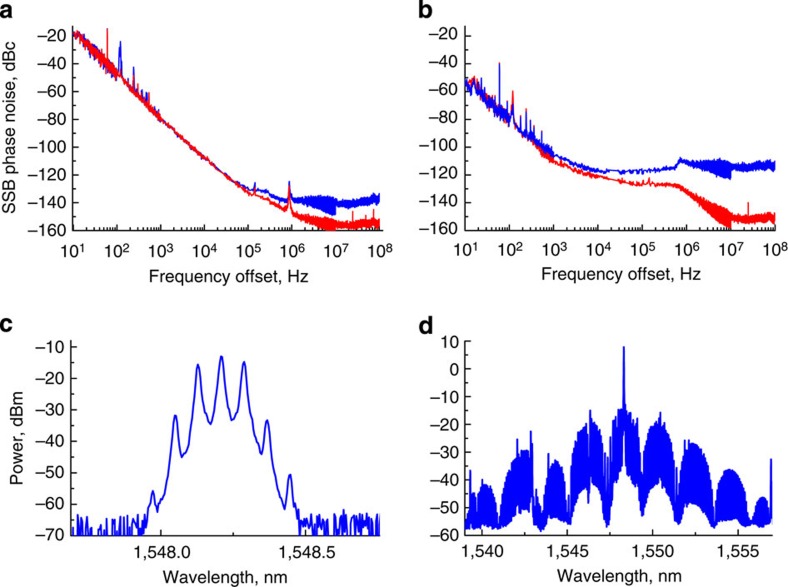
Phase noise of the RF signal generated by demodulation of a Kerr frequency comb on a fast PD. The RF signal is generated by light retrieved from the resonator using the input (blue line) and output (red line) evanescent field prism couplers. Phase noise measured in the case of a narrow (broad) frequency comb is shown in panel (**a**,**b**). The optical spectra of the narrow (broad) frequency combs are shown in panels (**c**,**d**), respectively.

In Fig. 4 the difference between phase noise levels obtained when the frequency comb is retrieved from the input and output prism couplers can be seen. The reason for the phase noise reduction is the filtering action of the resonator stripping off the noisy time independent (DC) background from the comb signal. This noise is from the pump laser and is present at the input coupling prism's output^45^. The excessive DC background is due to imperfect mode matching between the resonator and the pump light.

Phase noise of the signal retrieved through a second prism improves at offset frequencies exceeding the bandwidth of the mode, and the filtered signal reaches a shot noise limited floor, while the noise of the unfiltered signal is 30 dB higher.

The same figure shows the difference between the phase noise of RF signals generated by narrow and broad frequency combs. Comparing panels (a) and (b) of Fig. 4, we notice that the signal generated by the narrow comb has higher close-in phase noise and smaller noise floor, while the RF signal from the broad comb has lower close-in noise and higher noise floor. This result qualitatively confirms our earlier theoretical predictions^46^.

**Figure 5 f5:**
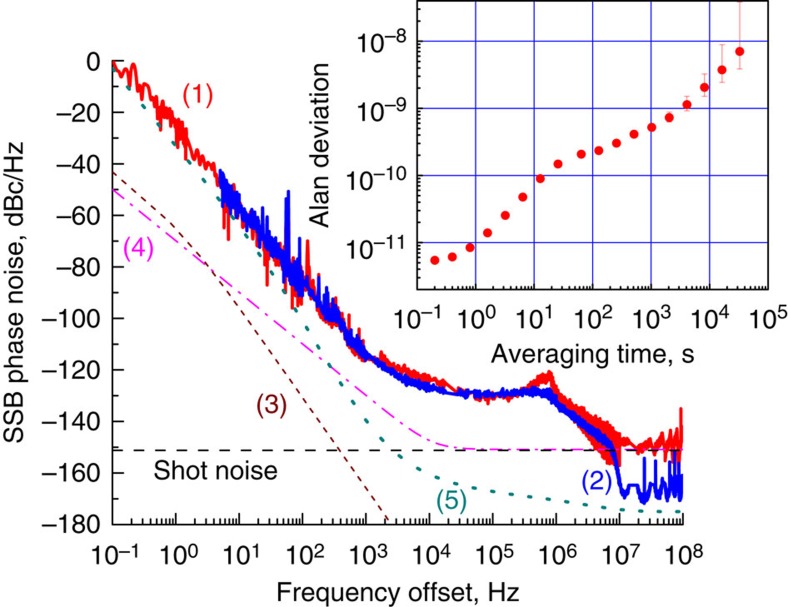
Phase noise of the 10 GHz RF signal generated by the wide Kerr frequency comb without (red line, (1)) and with (blue line, (2)) a narrow-band RF filter placed after the PD. The measured noise at offset frequencies below 1 kHz and above 10 MHz are within 3 dB of the noise floor of the OEwaves microwave phase noise measurement system used. Curves (3) and (4) describe theoretically found fundamental thermorefractive^50^ and quantum noise^46^ of the oscillator, respectively. Sensitivity of our PNTS is described by curve (5). The inset shows Allan deviation of the RF signal. Spectrum of the Kerr frequency comb resembles the optical spectrum shown in inset of Fig. 3.

We found that the noise floor of the signal can be further reduced if an RF bandpass filter is inserted after the PD Fig. 5. This is always the case when the shot noise of the signal exceeds the thermal noise limit. The observed close-in noise as well as the noise floor (Fig. 5) is nearly at the limit of the floor of the microwave phase noise measurement system we used. A low frequency beat from two identical comb oscillators must be used for a more accurate measurement of the noise with low phase noise measurment systems that have lower floor.

### Study of spectral purity of the RF signal

A typical phase noise spectrum resulting from a broad-band optical oscillation (Fig. 5) has three distinct features. Close-in noise that varies as *f*^−3^; a noise ‘plateau' in 1 kHz–1 MHz frequency range that has a complex quasi-resonant frequency dependence; and a noise floor at frequencies exceeding 10 MHz. We attribute the close-in noise to the thermal noise of the resonator due to the intensity noise of the laser and performed two experiments to validate this assertion (see Discussion section). The noise plateau is also the result of the transfer of the laser amplitude noise to the phase noise of the RF signal, but through intrinsic phase-locking mechanism of the comb, a different mechanism from the thermal effect. The noise floor is given by the detector shot noise and depends on the power of the RF signal at the output of the PD.

The phase noise spectrum shown in Fig. 5 is already excellent for a 10 GHz oscillator. However, it can be improved even more, if we compare it with the ultimate limitations resulting from fundamental physics. Quantum physics teaches us that any oscillator has an unavoidable noise resulting from quantum vacuum fluctuations. The origin of this noise is analogous to phase diffusion of a laser which impacts its linewidth. The quantum noise evaluated for the Kerr frequency comb^46^ leads to the limit depicted by line (4) in Fig. 5. The spectral purity of the oscillator is also limited owing to thermodynamic fluctuations of the resonator temperature and volume, resulting in line (3) in Fig. 5. We do not see any reason why the fundamental limits cannot be reached in even more refined Kerr comb RF photonic oscillators. To achieve such an outstanding performance the pump laser intensity noise has to be reduced by at least two orders of magnitude and the power of the RF signal has to be increased. The intensity noise can be reduced by improved thermal management of the laser chip as well as by improvement of the laser current driver. The RF power can be increased by integrating a more powerful diode laser with the nonlinear resonator.

## Discussion

An important question regarding the performance of the oscillator described above is what limits it. To answer this we performed several experiments.

### Optical frequency and repetition rate of the comb oscillator

**Figure 6:**
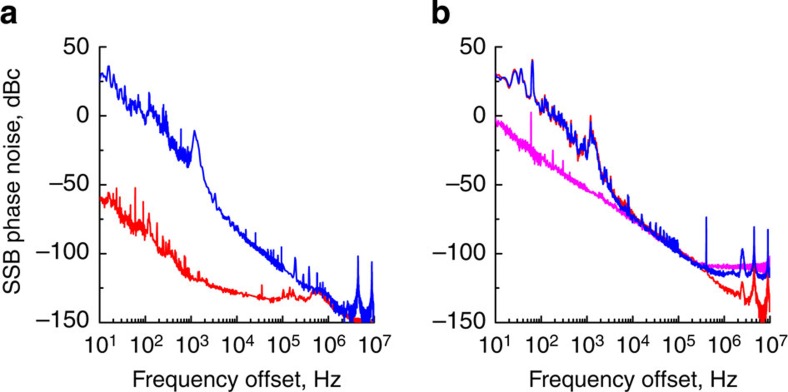
Sources of the oscillator noise. (**a**) A comparison between the phase noises of an injection locked DFB laser (blue) and the RF signal generated by the Kerr comb (red). The spurs at offset frequencies smaller than 1 kHz are from the acoustic noise picked up by the fiber interferometer used to measure the laser phase noise. The difference between the phase noise values is about 86 dB at close-in frequencies, which corresponds to 20log(*ν*_0_/*ν*_RF_). (**b**) Phase noise of two independent lasers locked to two modes belonging to different families of the same resonator, and the phase noise of the RF beat note of the lasers. Phase noise of the laser reflected from the resonator is shown by the blue line, phase noise of the laser transmitted through the resonator is shown by the red line, phase noise of the 6.5 GHz beat note of the lasers is shown by the magenta line. Comparing the noise floor of the red and blue lines we find another confirmation of the reduction of the laser phase noise when add-drop coupler configuration is used. The RF beat note has a higher noise floor since one of the lasers has higher noise. Both lasers have the same close-in phase noise determined by the frequency jitter of the resonator modes. The RF beat note has smaller noise since the common noise contribution resulting from the jitter is removed. The noise of the RF signal reflects self-injection locking of the lasers and corresponding resonator modes. It is comprised of the frequency noise of the detuning of the laser frequency and frequency of the corresponding optical mode.

To measure phase noise of the distributed feedback (DFB) laser we used an optical phase noise test system (optical PNTS) developed at OEwaves. During this measurement we ensured that (i) the laser was self-injection locked by means of the WGM resonator^13^, and (ii) that Kerr frequency comb was generated in the WGM resonator. The measured phase noise of the laser is compered in (Fig. 6a) with the measured phase noise of the RF signal produced by the Kerr frequency comb on a fast PD. At small offset frequencies the difference between these two curves is about 86 dB. This is 20 times logarithm of the ratio between the optical frequency (*ν*_0_=200 THz) and the free spectral range (FSR) of the resonator coinciding with the frequency comb repetition rate (*ν*_RF_=9.9 GHz). This comparison points out that the phase noise of the Kerr frequency comb RF oscillator and phase noise of the pump laser are both limited by the fluctuations of the resonator frequency.

The reason for this correlation is the thermal fluctuations of the resonator that determine both fluctuations of the laser frequency (the laser is locked to a WGM) and the comb repetition rate. Variation of temperature of the resonator resulting from environmental fluctuations, change of the laser frequency and power, as well as change of any strain on the resonator resulting from the bonding structures, modify the optical frequency of the modes and FSR of the resonator. These changes have a degree of correlation resulting in the observed ratio of the phase noise of the pump light and the frequency comb repetition rate.

### Technical noise sources

To confirm this observation we assembled another test set-up with two identical lasers coupled to a single WGM resonator. One of the lasers was self-injection locked to one resonator mode and the other—to another resonator mode belonging to a different mode family. This was done to avoid any possible cross-talk between the lasers via resonant four wave mixing. The modes used were separated by 6.5 GHz, while the resonator FSR was 35 GHz. The laser power was reduced so that neither thermal nor optical nonlinear effects were significant in the system. We measured the phase noise of the lasers individually, and then measured the phase noise of the their beat note [Fig f2][Fig f3][Fig f4][Fig f5](Fig. 6b). The measurement shows that the close-in phase noise of each laser exhibits a *f*^−3^ frequency dependence, while the beat note of the lasers has a ≈*f*^−2^ frequency dependence. Since the lasers are mutually incoherent, we conclude that the close-in laser noise is due to the noise of the resonator, while the beat note illustrates the locking stability of the lasers to the resonator modes. The noise of the resonator, in turn, results from transduction of the technical noise in the set-up. This technical noise is dominated by the thermal noise associated with the laser relative intensity noise, if the laser power is large enough.

**Figure 7:**
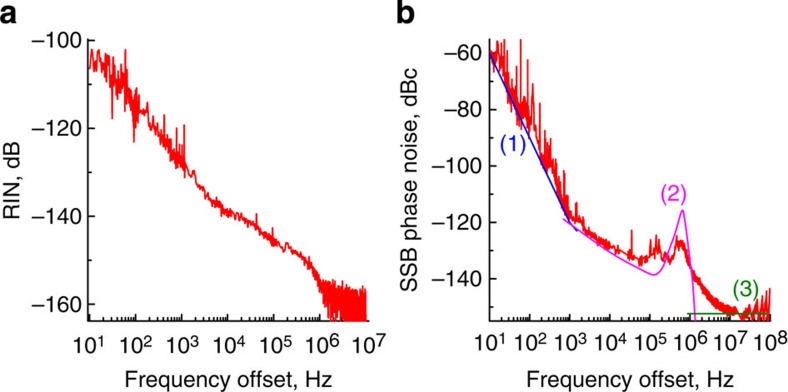
Impact of the relative intensity noise (RIN) of the pump laser on the oscillator performance. (**a**) RIN of the pump laser. (**b**) Theoretical fit (blue, (1), magenta, (2) and green, (3), lines) of the experimental data for the comb phase noise (red line). The close-in noise, (1), is selected to be resulting from the division of the resonator thermal noise, for example, noise associated with the laser RIN. The noise floor, (3), is assumed to be shot noise limited. The rest of the theoretical curve, (2), results from the transfer of the laser amplitude noise, *P*_DC_ × RIN(*f*), to the RF phase noise. The transfer function is simulated numerically.

To explain the phase noise at intermediate frequencies we performed numerical simulation using ideal parameters of the WGM resonator. In this numerical model we neglect fluctuations of all kinds including those of the resonator temperature. We modulated the amplitude of the cw pump at different frequencies, evaluated the RF signal produced by the comb on a PD, and determined the frequency dependent transfer function of the amplitude modulation of light to the phase modulation of the RF signal produced by the comb. Then we measured the amplitude noise of the pump light (Fig. 7a) and multiplied it by the transfer function. The resultant prediction of the phase noise is shown in Fig. 7b^47^.

### Phase noise floor

Finally, to calculate the phase noise floor we used:


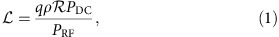


where *q* is the charge of electron, *ρ*=50 Ω is the resistance of the PD load, 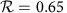 A W^−1^ is responsivity of the PD, *P*_DC_=5 mW is the DC optical power reaching the PD, *P*_RF_=50 μW is the power of the 10 GHz signal generated by the comb. Using these numbers we find 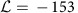 dBc Hz^−1^, which matches the phase noise floor of the oscillator

## Conclusion

A stable and spectrally pure X-band RF photonic oscillator is demonstrated experimentally. The phase noise of the 9.9 GHz signal produced by the oscillator is less than −60 dBc Hz^−1^ at 10 Hz frequency offset and −120 dBc Hz^−1^ at 1 kHz frequency offset. The Allan deviation of the signal is 10^−11^ at 1 s integration. The oscillator is based on a Kerr optical frequency comb excited in a high-Q magnesium fluoride WGM resonator. At small offset frequencies the phase noise of the RF signal produced by the oscillator is correlated with the phase noise of the laser light exciting the resonator. This noise originates from the fluctuations of the frequency of the resonator. Hence, further thermal and mechanical stabilization of the resonator and the laser will result in improvement of the RF phase noise. Improvement of the higher frequency noise is also possible if lasers with smaller amplitude fluctuations and higher power are used for resonator pumping.

## Methods

**Figure 8:**
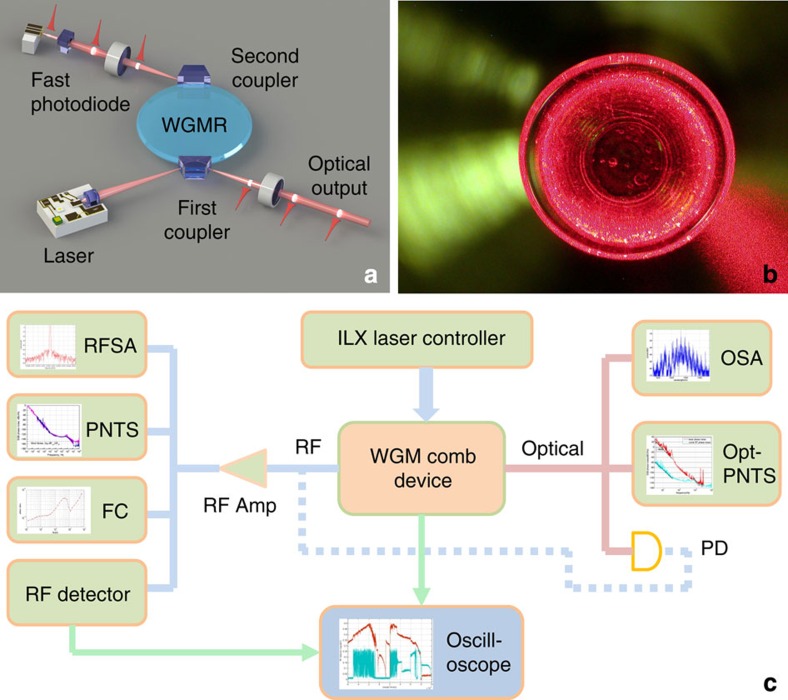
Experimental set-up. (**a**) Schematic diagram of the set-up of the RF photonic oscillator based on optical hyper-parametric oscillation in a MgF_2_ crystalline WGM resonator. The resonator is pumped with a semiconductor DFB laser using an evanescent wave prism coupler (first coupler, add port). The optical frequency comb generated in the resonator leaves it via the first and the second (drop port) prism couplers. Demodulating the light leaving the second prism on a fast PD, which is integrated in the oscillator package, generates spectrally pure RF signals at frequencies corresponding to multiples of the FSR of the resonator. (**b**) A WGM resonator with high order modes visualized with red light. (**c**) RF photonic oscillator test set-up. The RF signal generated by the frequency comb is analyzed using an RF spectrum analyzer, microwave PNTS, frequency counter and RF power detector. The light leaving the first coupler is sent to an optical spectrum analyzer and optical PNTS or another PD. The output of this PD (shown by a dashed line) is analyzed in the same way as the output of the PD integrated with the set-up.

A schematics of the Kerr frequency comb-based RF photonic oscillator set-up is illustrated by Fig. 8. A cylindrical crystalline magnesium fluoride preform is used to fabricate a WGM optical resonator by mechanical polishing. The resonator is 0.1 mm in thickness and 7.1 mm in diameter. These geometrical dimensions correspond to 9.9 GHz FSR of the fundamental mode family of the resonator. We found that the intrinsic (unloaded) full width at the half maximum of the modes is 35 kHz.

Evanescent field prism couplers are used to interrogate the resonator modes. The corresponding loaded (with two prism couplers) full width at the half maximum bandwidth of the modes approaches 300 kHz. A semiconductor DFB laser is coupled to the resonator through the input prism. A portion of the light is backscattered to the laser due to resonant Rayleigh scattering inducing self-injection locking of the laser frequency to the frequency of a selected resonator mode. This self-injection locking effect^13^, results in 30–40 dB improvement of the laser linewidth, if compared with the linewidth of the free running DFB laser, which is necessary to efficiently couple light from the laser to a high-Q WGM (the laser has to have narrower linewidth than the bandwidth of the WGM). The locking mechanism is stable even though the nonlinear process associated with Kerr frequency comb generation modifies the resonant response of the mode rather significantly. The locking also keeps the oscillator running through the variations of temperature of the oscillator platform. The maximum optical power sent to the resonator is <25 mW. The optical power collected at the PD is 5 mW.

The frequency of self-injection locked laser does not drift much and the system stays locked for many days, provided that the temperature of the whole build is stabilized at 0.1 K level. The detuning between the laser and the pumped WGM drifts within the bandwidth of the mode. The maximum magnitude of the drift is reduced with improvement of thermal stabilization of the oscillator unit. We were able to achieve stable operation for tens of hours. Future improvement of the oscillator requires tightening the lock to avoid thermal effects resulting from the wandering of the laser frequency within the mode bandwidth.

The Kerr frequency comb is generated when the power of the circulating light in the pumped mode exceeds a certain threshold. We noticed, that depending on the particular mode selected and the loaded Q of this mode, the threshold optical power may vary from hundreds of μW to a few mW. The reason for the optical harmonics to appear is the modulation instability of the cw field confined in the mode of the nonlinear resonator. The individual optical harmonics of the coherent Kerr frequency comb have approximately the same linewidth as the linewidth of the pump light. However, their frequency fluctuations are strongly correlated. Beating of the optical comb signal leaving the resonator on a PD results in generation of a spectrally pure RF signal with linewidth orders of magnitude smaller than the linewidth of each optical harmonic.

The RF signal at the output of the fast PD was amplified with an electric amplifier, and then distributed to various measurement devices. The spectra of the RF signal was measured using an Agilent E4446A RF spectrum analyzer. The phase noise was measured with an OEwaves microwave PNTS. The frequency stability was characterized with a HP5372A frequency counter, stabilized with a Rb clock Stanford Research Systems (SRS). The power level of the RF signal was measured using a RF detector and monitored on the oscilloscope.

The optical signal exiting the first prism coupler was coupled into a fiber and sent to the optical spectrum analyzer (Ando 6317B) for capturing the spectra of the generated Kerr comb, and to OEwaves optical PNTS for measuring the phase noise of the injection locked pump DFB laser. The optical signal was also sent to a fast fiber coupled Discovery PD. The RF beat signal generated by this PD was compared with the RF signal generated by another PD, which received the optical signal of the drop port of the resonator.

## Additional information

**How to cite this article:** Liang, W. *et al.* High spectral purity Kerr frequency comb radio frequency photonic oscillator. *Nat. Commun.* 6:7957 doi: 10.1038/ncomms8957 (2015).

## Figures and Tables

**Figure 1:**
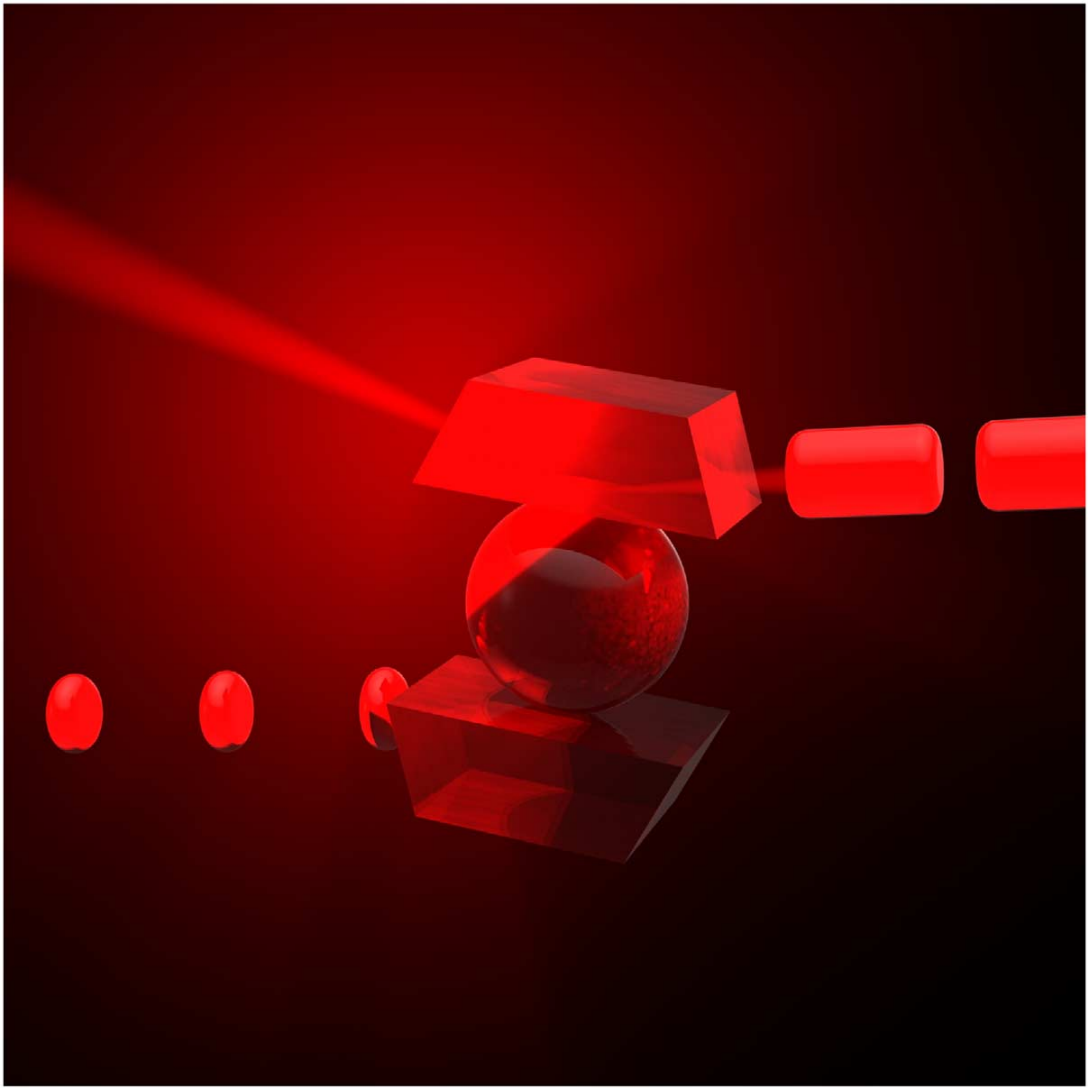
A conceptual depiction of a Kerr frequency comb oscillator consisting of a spherical nonlinear microcavity integrated with two evanescent field prism couplers. The cavity, pumped with cw light through the input prism, generates amplitude modulated light, ultimately represented by a train of dark and bright optical pulses. The dark pulses are leaving the input prism while bright pulses are exiting the output prism^46^. The pulses exiting either prism coupler, generate a spectrally pure RF signal at the output of a fast photodetector. The phase noise produced by the train of bright pulses is usually lower since the resonator filters out the classical noise associated with the pump laser.
